# The Impact of Neck Dissection on the Functional Integrity of Spinal Accessory Nerve in Head and Neck Cancers

**DOI:** 10.7759/cureus.105402

**Published:** 2026-03-17

**Authors:** Sandeepan Bhattacharya, Vikas Gupta, Utkal P Mishra, Ganakalyan Behera, Ruchi Singh, Rajesh Malik

**Affiliations:** 1 Otolaryngology - Head and Neck Surgery, All India Institute of Medical Sciences, Bhopal, Bhopal, IND; 2 Physiology, All India Institute of Medical Sciences, Bhopal, Bhopal, IND; 3 Radiodiagnosis, All India Institute of Medical Sciences, Bhopal, Bhopal, IND

**Keywords:** accessory nerve, electromyography, head and neck neoplasms, intraoperative neuromonitoring, neck dissection, shoulder dysfunction

## Abstract

Background and objective

Neck dissection remains a cornerstone in the management of head and neck cancers with cervical lymph node metastasis. Despite advances in nerve-sparing surgical techniques, shoulder dysfunction remains a recognized complication following neck dissection. This study aimed to evaluate whether anatomical preservation of the spinal accessory nerve (SAN) correlates with its functional integrity and postoperative shoulder function following neck dissection in head and neck cancer patients.

Methods

This prospective, longitudinal observational study enrolled 25 patients undergoing selective neck dissection (SND) or modified radical neck dissection (MRND) for biopsy-proven head and neck malignancies. Preoperative and postoperative assessments conducted at two and four weeks included clinical shoulder examination, goniometric range of motion (ROM), Shoulder Pain and Disability Index (SPADI), and electromyography (EMG) of the trapezius muscle. Radiological assessment of sternocleidomastoid (SCM) and trapezius muscle volume was performed preoperatively and at six months. Intraoperative nerve monitoring (IONM) was employed in all cases.

Results

All patients had normal preoperative shoulder function. At two weeks postoperatively, significant deterioration was observed in shoulder abduction, shrug strength, cervical rotation, and SPADI scores (p < 0.001). Partial recovery was noted at four weeks; however, deficits persisted in a subset of patients. Postoperative EMG demonstrated significant changes on the operated side (p = 0.031). At six months, a statistically significant reduction in the trapezius muscle volume was observed (p < 0.001), with a greater percentage reduction in volume in the MRND group compared to the SND group. IONM parameters did not significantly differ between dissection types and did not consistently correlate with postoperative functional outcomes.

Conclusions

Anatomical preservation of the SAN does not necessarily ensure functional integrity. Early postoperative shoulder dysfunction occurs even in nerve-sparing procedures, with more pronounced structural changes following a modified radical neck dissection. Multimodal postoperative assessment and early rehabilitation are essential. Larger studies with longer follow-up periods are warranted to further clarify the prognostic role of IONM.

## Introduction

Neck dissection is an integral part of the management of head and neck malignancies. Despite considerable advancements in surgical techniques and increasing emphasis on the preservation of non-lymphatic structures, postoperative functional morbidity remains a major clinical concern. Among these structures, the spinal accessory nerve (SAN) plays a vital role in shoulder stability and movement by innervating the trapezius muscle. Injury to the SAN can lead to shoulder pain, weakness, limited abduction, scapular winging, and functional disability, significantly affecting postoperative quality of life [1].

Historically, radical neck dissection included the routine sacrifice of the SAN, leading to a high incidence of shoulder dysfunction. To reduce this morbidity, modified radical neck dissection (MRND) and selective neck dissection (SND) were developed, both of which aim to achieve anatomical preservation of the SAN. Although these techniques have significantly reduced the incidence of overt nerve injury, numerous patients continue to experience postoperative shoulder dysfunction despite confirmed anatomical preservation of the nerve. This suggests that anatomical preservation alone may not accurately represent functional nerve integrity [2,3].

Several mechanisms have been proposed to explain postoperative SAN dysfunction in anatomically preserved nerves, including traction injury, devascularization, thermal damage, compression, and postoperative fibrosis [3]. Such subclinical injuries may impair nerve conduction and muscle function without clear intraoperative evidence of nerve damage. Electrophysiological studies using electromyography (EMG) have demonstrated changes in trapezius muscle activity in patients with preserved SANs, correlating with clinical shoulder impairment [4]. Current literature primarily focuses on clinical assessment of the shoulder or patient-reported outcomes, with limited studies that integrate objective electrophysiological assessments and radiological evaluation of muscle integrity [5,6]. Furthermore, the correlation between intraoperative nerve preservation, postoperative functional status, and structural changes in the trapezius muscle remains poorly understood.

This study was designed to assess the impact of neck dissection on the functional integrity of the SAN even when it is anatomically preserved. Using a multimodal assessment that included clinical shoulder examination with range-of-motion (ROM) analysis, patient-reported disability scores (SPADI), EMG, radiological muscle volume evaluation, and intraoperative nerve monitoring (IONM), we sought to determine whether anatomical preservation of the SAN corresponds to functional preservation following selective and MRND in patients with head and neck malignancies. The primary objective was to assess the relationship between anatomical preservation of the SAN and postoperative shoulder function. Secondary objectives included evaluating electrophysiological and structural muscle changes, comparing outcomes between selective and MRND, and exploring the relationship between IONM parameters and postoperative functional outcomes.

## Materials and methods

This prospective, longitudinal, observational study was conducted over 18 months, from August 2020 to November 2021, in the Department of Otorhinolaryngology and Head & Neck Surgery at a tertiary care referral centre in central India. The study commenced after obtaining Institutional Human Ethics Committee approval, and written informed consent was obtained from all participants. The study population comprised patients attending the ENT outpatient department who were clinically diagnosed with biopsy-proven head and neck malignancies and subsequently underwent SND or MRND. Tumor staging was performed according to the eighth edition of the American Joint Committee on Cancer (AJCC) TNM staging system [7]. Patients with pre-existing spinal accessory nerve dysfunction or shoulder joint pathology on the operated side, diagnosed neuromuscular disorders, prior radiotherapy, those undergoing radical neck dissection, or requiring PMMC or deltopectoral flap reconstruction were excluded. A total of 30 patients were initially enrolled; however, five were lost to follow-up at six months and were excluded from the final analysis.

All patients underwent preoperative assessment of spinal accessory nerve function through detailed clinical examination, goniometric measurement of shoulder ROM, and patient-reported evaluation using the SPADI. All clinical assessments were performed by the same examiner using standardized clinical examination techniques. Shoulder shrug strength was assessed against manual resistance, and shoulder symmetry was evaluated through visual inspection of shoulder droop and scapular positioning during rest and movement. SPADI is a freely available, validated, self-administered questionnaire used to assess shoulder pain and functional disability [8]. It consists of 13 items divided into two subscales: a five-item pain subscale and an eight-item disability subscale. Each item is scored using a visual analog scale or numerical rating scale, and the total score is expressed as a percentage, with higher scores indicating greater pain and disability. The SPADI questionnaire requires approximately five minutes to complete and has been widely used for the assessment of shoulder dysfunction in clinical and research settings.

Clinical assessment of shoulder function included evaluation of shoulder abduction, shoulder shrug with and without resistance, muscle strength, resting muscle tension, muscle length, cervical ROM, ipsilateral cervical flexion, contralateral cervical rotation, and cervical flexion on both operated and non-operated sides. Needle EMG of the trapezius muscle was performed preoperatively and at four weeks postoperatively using the Neuropack S3 electrophysiological system (Nihon Kohden Corporation, Tokyo, Japan). The recording needle electrode was placed in the upper trapezius muscle approximately midway between the cervical spine and the acromion. EMG findings were graded according to the scoring system described by Tsuji et al. [9]. This system evaluates trapezius muscle function on a 5-point scale based on spontaneous activity at rest, motor unit potential morphology during minimal contraction, and the interference pattern during maximal contraction. Lower grades indicate denervation with electrical silence or polyphasic motor unit potentials, whereas higher grades represent normal recruitment and interference patterns.

Radiological assessment of trapezius and sternocleidomastoid muscle volumes was performed using CT and MRI scans with Syngo.via software. Muscle boundaries were manually segmented on axial slices, and muscle volumes were calculated using three-dimensional reconstruction tools. Muscle volume ratios were calculated by comparing the operated and non-operated sides at the six-month follow-up.

**Figure 1:**
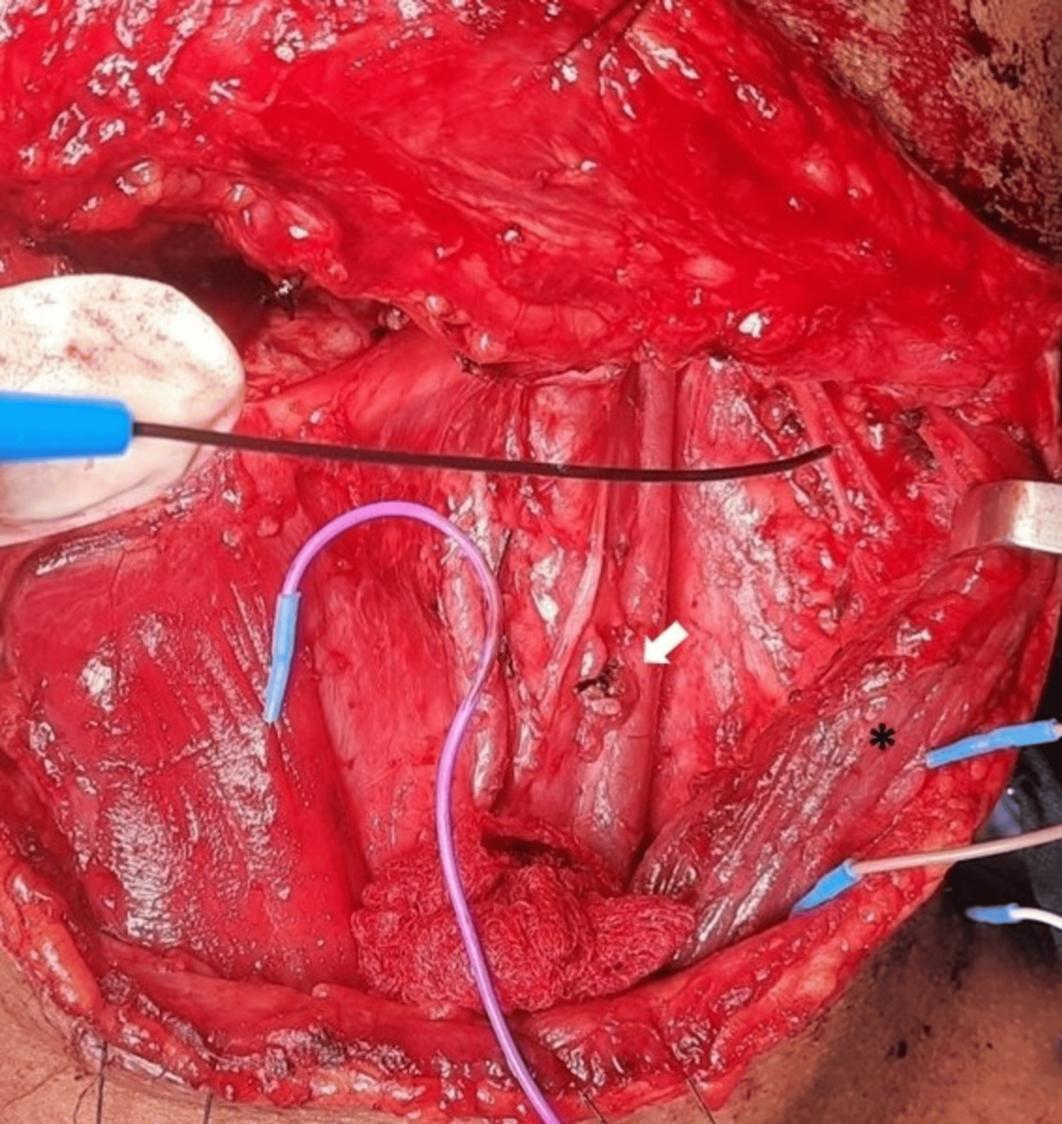
Intraoperative nerve monitoring of left spinal accessory nerve during neck dissection Nerve stimulator probe pointing towards the spinal accessory nerve. Black asterisk denotes retracted sternocleidomastoid muscle and white arrow denotes internal jugular vein.

All neck dissections were performed by a single head and neck surgeon experienced in neck dissections. Intraoperative spinal accessory nerve monitoring was performed in all cases using Xltek® Protektor32 stimulator (Natus Neurology Incorporated, Middleton, WI). Monopolar electrocautery was avoided near the spinal accessory nerve during level II and level V nodal dissections. Subdermal needle electrodes were placed in the trapezius, sternocleidomastoid, strap, and deltoid muscles. The spinal accessory nerve was stimulated with a monopolar probe approximately 1 cm proximal to its entry into the sternocleidomastoid muscle upon identification (Figure 1). Stimulus intensity was gradually increased until a reproducible electromyographic waveform was obtained, and threshold and amplitude values were recorded.

Measurements were repeated after neck dissection, and a threshold difference of ≥0.25 mA between initial and final recordings was considered significant. All patients were advised standardized postoperative shoulder mobilization and ROM exercises as part of the routine institutional rehabilitation protocol beginning in the early postoperative period. Postoperative evaluation was conducted at two and four weeks using the same clinical, electrophysiological, radiological, and patient-reported outcome measures. Functional outcomes were analyzed in relation to the type of neck dissection and IONM findings. All questionnaires, scoring systems, and classification systems used in this study are publicly available and free for academic research use.

Statistical analysis was performed using SPSS software version 29.0 (IBM Corp., Armonk, NY). Continuous variables were assessed for normality using the Shapiro-Wilk test. Normally distributed data were expressed as mean ± standard deviation (SD), while non-normally distributed data were expressed as median with interquartile range (IQR). Comparisons of repeated measurements (preoperative, two weeks, and four weeks) were performed using the Friedman test for non-parametric data. Paired comparisons between two related groups were analyzed using the Wilcoxon signed-rank test. Comparisons between independent groups (SND vs. MRND) were performed using the Mann-Whitney U test for continuous variables and the Chi-square or Fisher’s exact test for categorical variables, as appropriate. A p-value < 0.05 was considered statistically significant.

## Results

A total of 30 patients were initially enrolled in the study. Five patients were lost to follow-up at six months and were excluded, leaving 25 patients for the final analysis. Baseline demographic and clinical characteristics are summarized in Table 1. The mean age of participants was 42.28 ± 10.55 years, with a male predominance (76%). Squamous cell carcinoma constituted the majority of diagnoses (64%), with T2 being the most common tumor stage (48%). Most patients had N0 disease (60%). The right side was operated on in 60% of cases. SND was performed in 52% of cases, while MRND was performed in 28%. Primary closure was the most common reconstructive method (72%), and more than half of the patients received postoperative radiotherapy (56%).

**Table 1:** Demographic and clinical characteristics of the study population Tumor staging was performed according to the eighth edition of the American Joint Committee on Cancer (AJCC) TNM staging system [7] SD: standard deviation; SCC: squamous cell carcinoma, MRND: modified radical neck dissection; (m): metastatic

Variable	Value
Age, years, mean ± SD	42.3 ± 10.6
Sex, n (%)	
Male	19 (76%)
Female	6 (24%)
Operated side, n (%)	
Right	15 (60%)
Left	10 (40%)
Histopathological diagnosis, n (%)	
Moderately differentiated SCC	9 (36%)
Well-differentiated SCC	7 (28%)
Papillary carcinoma of the thyroid	4 (16%)
Adenoid cystic carcinoma	2 (8%)
Medullary carcinoma of the thyroid	1 (4%)
Metastatic adenocarcinoma	1 (4%)
Mucoepidermoid carcinoma	1 (4%)
T stage, n (%)	
T0 (occult primary)	1 (4%)
T1	4 (16%)
T2	12 (48%)
T3	4 (16%)
T4a	2 (8%)
T1b (m)	1 (4%)
T3a (m)	1 (4%)
N stage, n (%)	
N0	15 (60%)
N1a	2 (8%)
N1b	4 (16%)
N2a	1 (4%)
N2b	1 (4%)
N3b	2 (8%)
Primary surgical procedure, n (%)	
Wide local excision	12 (48%)
Total thyroidectomy	5 (20%)
Total parotidectomy	4 (16%)
Hemiglossectomy	3 (12%)
Radical submandibular gland excision	1 (4%)
Type of neck dissection, n (%)	
Selective neck dissection	13 (52%)
MRND type III	5 (20%)
MRND type II	2 (8%)
Central + lateral compartment neck dissection	4 (16%)
Central + posterolateral neck dissection	1 (4%)
Reconstruction, n (%)	
Primary closure	18 (72%)
Split-thickness skin graft (STSG)	4 (16%)
Abbe-Estlander flap	1 (4%)
Radial forearm free flap (RFFF)	1 (4%)
Submental flap	1 (4%)
Adjuvant therapy, n (%)	
Radiotherapy	14 (56%)
Chemoradiotherapy	1 (4%)
Radioiodine ablation	3 (12%)
None	7 (28%)

All patients demonstrated normal shoulder function on the operated side preoperatively. At two weeks postoperatively, significant deterioration in shoulder abduction and shrug strength was observed, with 64-68% of patients exhibiting reduced function (p < 0.001). Contralateral cervical rotation was decreased in 72% of patients at two weeks (p < 0.001). Shoulder and neck asymmetry were also frequently observed during the early postoperative period (Table 2). By four weeks, partial recovery was evident, with reductions in the proportions of patients demonstrating impaired shoulder abduction (32%) and shrug strength (24%). Contralateral cervical rotation impairment similarly decreased to 24%. Ipsilateral cervical flexion showed minimal postoperative change and did not reach statistical significance (p = 0.091). 

**Table 2:** Comparison of pre-op and post-op shoulder function in study participants Data presented as n (%). Statistical comparison performed using Cochran’s Q test for repeated categorical measures. P < 0.05 is considered significant

Parameter	Preoperative	Two weeks post-op	Four weeks post-op	Cochran’s Q, p-value
Shoulder abduction (without resistance)	0 (0%)	17 (68%)	8 (32%)	29.3, p < 0.001
Shoulder abduction (with resistance)	0 (0%)	10 (67%)	4 (27%)	21.5, p < 0.001
Shrug strength (with resistance)	0 (0%)	16 (64%)	6 (24%)	27.4, p < 0.001
Shrug strength (without resistance)	0 (0%)	16 (64%)	6 (24%)	27.4, p < 0.001
Contralateral cervical rotation	0 (0%)	18 (72%)	6 (24%)	30.6, p < 0.001
Ipsilateral cervical flexion	0 (0%)	4 (16%)	1 (4%)	4.80, p = 0.091
Shoulder asymmetry at rest	0 (0%)	17 (68%)	8 (32%)	29.3, p < 0.001
Neck asymmetry at rest	0 (0%)	14 (56%)	7 (28%)	22.1, p < 0.001

Shoulder ROM on the operated side demonstrated significant variation across follow-up periods (Table 3). Shoulder flexion declined significantly at two weeks postoperatively, followed by improvement at four weeks (p < 0.001). Similarly, shoulder abduction was reduced considerably at two weeks and partially recovered by four weeks (p = 0.020). In contrast, shoulder extension, internal rotation, and external rotation did not demonstrate statistically significant changes during follow-up.

**Table 3:** Comparison of range of motion of operated side shoulder joint in post operative period Data presented as median (IQR). Comparison performed using the Friedman test for repeated measures. P < 0.05 is considered significant IQR: interquartile range

Parameter	Preoperative	Two weeks post-op	Four weeks post-op	Friedman χ², p-value
Shoulder flexion	180 (180–180)	170 (150–180)	180 (170–180)	χ² = 15.6, p < 0.001
Shoulder extension	50 (45–55)	50 (44–55)	50 (45–55)	χ² = 0.18, p = 0.91
Shoulder internal rotation	90 (90–90)	90 (90–90)	90 (90–90)	χ² = 1.83, p = 0.40
Shoulder external rotation	90 (90–90)	90 (90–90)	90 (90–90)	χ² = 3.20, p = 0.20
Shoulder abduction	180 (180–180)	165 (140–176)	172 (154–180)	χ² = 7.82, p = 0.020

SPADI scores on the operated side showed significant increases in pain, disability, and total scores at two weeks postoperatively, followed by significant improvement at four weeks (Table 4). SPADI scores on the non-operated side remained unchanged throughout the follow-up period and are therefore not shown.

**Table 4:** Comparison of patient-reported SPADI scores across various domains during follow-up* ^*^[8] Data presented as median (IQR). Statistical comparison performed using the Friedman test. P < 0.05 is considered significant SPADI: Shoulder Pain and Disability Index; IQR: interquartile range

Characteristic	Preoperative	Two weeks post-op	Four weeks post-op	Friedman χ², p-value
SPADI pain score	0 (0–0)	23 (10–36)	0 (0–8)	χ² = 30.5, p < 0.001
SPADI disability score	0 (0–0)	0 (0–19)	0 (0–5)	χ² = 18.7, p < 0.001
SPADI total score	0 (0–0)	14 (5–32)	0 (0–8)	χ² = 29.2, p < 0.001

Electrophysiological and radiological muscle volume measurements revealed significant postoperative alterations in the operated side despite anatomical preservation of the spinal accessory nerve. At four weeks, trapezius EMG scores showed a statistically significant difference compared to preoperative values (p = 0.031), indicating early electrophysiological compromise in a subset of patients (Table 5). Furthermore, evaluation of muscle volume at six months post-op revealed a significant postoperative alteration in sternocleidomastoid muscle volume (p = 0.012). In contrast, trapezius muscle volume exhibited a highly significant reduction (p < 0.001) (Table 6).

**Table 5:** EMG assessments of trapezius muscle using the EMG scoring system described by Tsuji et al.* ^*^[9] Data presented as median (IQR). Electromyographic grading was performed according to the scoring system described by Tsuji et al. [9]. Statistical comparison performed using the Wilcoxon signed-rank test. P < 0.05 is considered significant EMG: electromyography; IQR: interquartile range

Parameter	Preoperative	Four weeks post-op	Wilcoxon Z, p-value
EMG score of trapezius (Operated side)	5 (5–5)	5 (5–5)	Z = −2.16, p = 0.031
EMG score of trapezius (Non-operated side)	5 (5–5)	5 (5–5)	Z = −0.22, p = 0.82

**Table 6:** Comparison of muscle volume at the six-month follow-up Data presented as median (IQR). Statistical comparison performed using the Wilcoxon signed-rank test. P < 0.05 is considered significant SCM: sternocleidomastoid muscle; IQR: interquartile range

Parameter	Preoperative	Six months post-op	Wilcoxon Z, p-value
SCM volume (operated side)	48 (42–55)	50.0 (45.6–53.4)	Z = −2.52, p = 0.012
Trapezius volume (operated side)	190 (173–202)	187 (172–201)	Z = −3.75, p < 0.001

Comparison between MRND and SND revealed no statistically significant differences in early shoulder range of motion or SPADI scores at two and four weeks postoperatively (Table 7). Although MRND patients demonstrated numerically higher early SPADI scores, these differences did not reach statistical significance.

**Table 7:** Comparison between SND and MRND SPADI sores are expressed in three domains [8]. EMG scores are obtained using the EMG scoring system described by Tsuji et al. [9]. Data presented as median (IQR). Statistical comparison performed using the Mann-Whitney U test (Wilcoxon rank-sum test). P < 0.05 is considered statistically significant SND: selective neck dissection; MRND: modified radical neck dissection; ROM: range of motion, SPADI: Shoulder Pain and Disability Index, EMG: electromyography; SCM: sternocleidomastoid; IQR: interquartile range

Outcome	MRND (n = 7)	SND (n = 18)	Mann-Whitney U, p-value
ROM at 2 weeks			
Shoulder abduction (degrees)	180 (178–180)	180 (174–180)	U = 58.0, p = 0.80
Shoulder flexion (degrees)	180 (180–180)	180 (170–180)	U = 51.5, p = 0.30
Shoulder extension (degrees)	40 (40–48)	48 (40–54)	U = 49.0, p = 0.40
ROM at 4 weeks			
Shoulder abduction (degrees)	180 (180–180)	180 (180–180)	U = 60.0, p = 0.60
Shoulder extension (degrees)	55 (50–60)	50 (50–55)	U = 52.0, p = 0.40
SPADI at 2 weeks (operated side)			
Pain score	12 (0–16)	0 (0–9)	U = 42.5, p = 0.20
Total score	14 (0–20)	0 (0–13)	U = 45.0, p = 0.30
Disability score	0 (0–6)	0 (0–0)	U = 47.0, p = 0.40
SPADI at 4 weeks (operated side)			
Pain score	7 (6–37)	8 (5–30)	U = 62.0, p > 0.90
Total score	14 (12–44)	10 (8–28)	U = 53.5, p = 0.60
Disability score	0 (0–19)	5 (0–20)	U = 55.0, p = 0.70
Electrophysiological assessment (4 weeks)			
EMG score	4 (3–4)	5 (5–5)	U = 19.0, p = 0.004
Muscle volume reduction (%)			
SCM volume reduction	7.8 (6.3–8.2)	1.0 (0–2.1)	U = 11.0, p = 0.002
Trapezius volume reduction	2.7 (0.5–3.3)	1.1 (0–2.1)	U = 27.0, p = 0.041

However, significant differences were observed in electrophysiological and structural outcomes. Postoperative EMG scores at four weeks were significantly lower in the MRND group compared to SND (p = 0.004). Additionally, percentage reduction in sternocleidomastoid muscle volume was substantially greater in MRND (7.8% vs. 1.0%, p = 0.002), as was trapezius muscle volume reduction (2.7% vs. 1.1%, p = 0.041).

Comparison of intraoperative nerve-monitoring parameters between SND and MRND showed no statistically significant differences in baseline threshold or amplitude values (Table 8). No statistically significant differences were observed between SND and MRND groups in terms of intraoperative amplitude or stimulation threshold values, either at nerve identification or at completion of neck dissection (p > 0.05). Although the mean change in stimulation threshold differed between groups, with an increase observed in the SND group and a reduction in the MRND group, this difference did not reach statistical significance (p = 0.09).

**Table 8:** Comparison of IONM parameters between types of neck dissection Stimulus threshold was recorded in mA, while EMG amplitude responses were recorded in mV. Data presented as mean ± SD. Statistical comparison performed using the Mann-Whitney U test. P < 0.05 is considered statistically significant IONM: intraoperative nerve monitoring; SND: selective neck dissection; MRND: modified radical neck dissection; SD: standard deviation

IONM variable	SND (n = 18)	MRND (n = 7)	Mann-Whitney U, p-value
Threshold on identification of nerve (mA)	189.25 ± 281.03	219.96 ± 347.71	U = 59.0, p = 0.81
Threshold after completion of neck dissection (mA)	351.31 ± 358.56	168.42 ± 183.61	U = 47.5, p = 0.23
Mean difference of threshold (mA)	162.06 ± 256.67	−51.54 ± 176.03	U = 35.0, p = 0.09
Amplitude on identification of nerve (mV)	0.708 ± 0.307	0.516 ± 0.245	U = 41.0, p = 0.16
Amplitude after completion of neck dissection (mV)	0.698 ± 0.267	0.679 ± 0.280	U = 61.0, p = 0.88
Mean difference of amplitude (mV)	−0.01 ± 0.274	0.163 ± 0.265	U = 39.0, p = 0.18

## Discussion

Shoulder dysfunction remains one of the most frequently reported complications following neck dissection. It is commonly characterized by shoulder pain, drooping, weakness of the shoulder shrug, and reduced ROM during abduction and flexion movements. In this study, we evaluated shoulder function using a multimodal approach: clinical exams, ROM measurements, patient-reported SPADI scores, EMG assessments, radiological analysis of muscle volume, and IONM. This comprehensive strategy gave us a clear picture of both the functional and structural health of the SAN. In our cohort, deterioration in shoulder abduction, shrug strength, and cervical rotation was observed at two weeks postoperatively, with partial recovery by four weeks. These findings are consistent with a recent systematic review, which reported that postoperative shoulder dysfunction remains prevalent even after nerve-sparing procedures, particularly in the early postoperative period [10]. Cheng et al. and Sobol et al. also demonstrated objective reductions in shoulder ROM following neck dissection, with severity correlating to the extent of surgical dissection [11,12].

Although SAN preservation has been shown to reduce morbidity in selective neck dissection compared to radical neck dissection, it does not eliminate dysfunction. Short et al. demonstrated improved outcomes when the SAN was preserved, but residual shoulder impairment persisted in a subset of patients [13]. Leipzig et al. described the classic “shoulder syndrome,” emphasizing that even modified and selective dissections may result in clinically relevant dysfunction. In this study, we also observed a functional decline of shoulder movement despite anatomical preservation of the SAN [14].

Electrophysiological evaluation in our study demonstrated a significant postoperative decline in trapezius EMG scores. This aligns with the findings of Tsuji et al., who reported EMG abnormalities following selective neck dissection despite gross nerve preservation [9]. Lima et al. further emphasized that SAN neuropathy may be detected electrophysiologically even when anatomical continuity is maintained [15]. Veyseller et al. similarly identified subclinical electrophysiological changes following functional neck dissection [16]. Notably, in our study, EMG deterioration was significantly greater in the MRND group compared to the SND group, supporting the observation by Cheng et al. that more extensive dissections are associated with greater functional compromise [11].

Radiological analysis of muscle volume revealed significant postoperative structural changes, particularly in MRND patients. Cho et al. proposed trapezius muscle volume measurement as a novel objective tool to assess shoulder dysfunction, demonstrating its association with postoperative morbidity [17]. Our findings extend this concept by evaluating both trapezius and sternocleidomastoid muscle volumes and calculating the percentage volume reduction. The significantly greater percentage reduction observed in MRND patients suggests that structural muscle atrophy parallels electrophysiological compromise and reflects cumulative nerve stress during more extensive dissections.

Intraoperative spinal accessory nerve monitoring has been proposed as a valuable tool for predicting postoperative shoulder dysfunction. Ling et al. demonstrated that intraoperative changes in SAN threshold and amplitude were associated with subsequent shoulder dysfunction and may serve as early prognostic indicators [18]. In contrast, Witt et al. reported no significant electrophysiological difference between selective and modified neck dissection procedures, suggesting that intraoperative monitoring parameters may not consistently correlate with postoperative outcomes [19]. Similarly, Lee et al. highlighted the utility of intraoperative monitoring in reducing the incidence of shoulder syndrome but could not establish definitive quantitative thresholds that predict dysfunction [6].

In the present study, although differences in threshold change were observed between the SND and MRND groups, they did not reach statistical significance. This aligns closely with the findings of Witt et al., indicating that intraoperative parameters alone may not fully reflect the extent of postoperative functional impairment [19]. Our results suggest that while intraoperative nerve monitoring remains valuable for nerve identification and preservation, postoperative electrophysiological and structural assessments provide more reliable indicators of functional integrity.

Interestingly, while electrophysiological and radiological differences between MRND and SND were statistically significant in our study, patient-reported SPADI scores and early range-of-motion measurements did not consistently demonstrate substantial differences between the groups. Selcuk et al. suggested that shoulder dysfunction following neck dissection is multifactorial and may involve contributions from cervical plexus branches, soft-tissue dissection, and postoperative fibrosis, in addition to SAN injury [2,20]. This may explain the discrepancy between objective structural and electrophysiological findings and subjective functional perception in the early postoperative period.

Overall, our findings suggest that anatomical preservation of the spinal accessory nerve does not necessarily result in preserved functional integrity. Early postoperative SAN dysfunction may be attributed to traction injury, devascularization, and surgical manipulation, particularly in more extensive dissections such as MRND. By integrating clinical, patient-reported, electrophysiological, radiological, and intraoperative data, this study provides a comprehensive evaluation of SAN-related morbidity. We recommend proactive physiotherapy and targeted exercise programs in the early postoperative period to minimize postoperative shoulder dysfunction and enhance recovery.

Limitations

Although we attempted a comprehensive multimodal assessment of SAN function by integrating clinical examination, SPADI scores, EMG analysis, radiological muscle volume measurements, and IONM within a prospective design, certain limitations must be acknowledged. The relatively small sample size may limit the statistical power of subgroup comparisons and affect the generalizability of the findings. In addition, the study was conducted by a single surgeon, which ensured procedural consistency but may limit the external applicability of the results. The inclusion of heterogeneous tumor types and surgical indications may also introduce clinical variability influencing postoperative shoulder function. Furthermore, clinical functional follow-up was limited to the early postoperative period, and longer-term evaluation would provide deeper insights into the trajectory of electrophysiological recovery and structural muscle remodeling following neck dissection. Finally, early postoperative shoulder dysfunction may also be influenced by factors such as postoperative pain, soft-tissue dissection, cervical plexus involvement, and variability in rehabilitation, which could act as confounding factors independent of direct spinal accessory nerve injury.

## Conclusions

Shoulder dysfunction may occur following neck dissection despite anatomical preservation of the spinal accessory nerve with the use of intraoperative neuromonitoring. Early postoperative clinical and electrophysiological impairment were observed, along with a significant reduction in muscle volume in patients undergoing MRND. Intraoperative nerve monitoring parameters did not reliably predict postoperative functional outcomes. These findings highlight the importance of early functional assessment and rehabilitation, but larger studies with longer follow-up are needed to better understand long-term recovery.

## References

[1] van Wilgen CP, Dijkstra PU, van der Laan BF, Plukker JT, Roodenburg JL (2003). Shoulder complaints after neck dissection; is the spinal accessory nerve involved?. Br J Oral Maxillofac Surg.

[2] Selcuk A, Selcuk B, Bahar S, Dere H (2008). Shoulder function in various types of neck dissection. Role of spinal accessory nerve and cervical plexus preservation. Tumori.

[3] Orhan KS, Demirel T, Baslo B, Orhan EK, Yücel EA, Güldiken Y, Değer K (2007). Spinal accessory nerve function after neck dissections. J Laryngol Otol.

[4] Tarkan Ö, Tuncer Ü, Bozdemir H (2012). Clinical and electrophysiological evaluation of shoulder functions in spinal accessory nerve-preserving neck dissection. Turk J Med Sci.

[5] Popovski V, Benedetti A, Popovic-Monevska D, Grcev A, Stamatoski A, Zhivadinovik J (2017). Spinal accessory nerve preservation in modified neck dissections: surgical and functional outcomes. Acta Otorhinolaryngol Ital.

[6] Lee CH, Huang NC, Chen HC, Chen MK (2013). Minimizing shoulder syndrome with intra-operative spinal accessory nerve monitoring for neck dissection. Acta Otorhinolaryngol Ital.

[7] Amin MB, Greene FL, Edge SB (2017). The Eighth Edition AJCC Cancer Staging Manual: Continuing to build a bridge from a population-based to a more "personalized" approach to cancer staging. CA Cancer J Clin.

[8] Breckenridge JD, McAuley JH (2011). Shoulder Pain and Disability Index (SPADI). J Physiother.

[9] Tsuji T, Tanuma A, Onitsuka T, Ebihara M, Iida Y, Kimura A, Liu M (2007). Electromyographic findings after different selective neck dissections. Laryngoscope.

[10] Gane EM, Michaleff ZA, Cottrell MA, McPhail SM, Hatton AL, Panizza BJ, O'Leary SP (2017). Prevalence, incidence, and risk factors for shoulder and neck dysfunction after neck dissection: a systematic review. Eur J Surg Oncol.

[11] Cheng PT, Hao SP, Lin YH, Yeh AR (2000). Objective comparison of shoulder dysfunction after three neck dissection techniques. Ann Otol Rhinol Laryngol.

[12] Sobol S, Jensen C, Sawyer W, Costiloe P, Thong N (1985). Objective comparison of physical dysfunction after neck dissection. Am J Surg.

[13] Short SO, Kaplan JN, Laramore GE, Cummings CW (1984). Shoulder pain and function after neck dissection with or without preservation of the spinal accessory nerve. Am J Surg.

[14] Leipzig B, Suen JY, English JL, Barnes J, Hooper M (1983). Functional evaluation of the spinal accessory nerve after neck dissection. Am J Surg.

[15] Lima LP, Amar A, Lehn CN (2011). Spinal accessory nerve neuropathy following neck dissection. Braz J Otorhinolaryngol.

[16] Veyseller B, Aksoy F, Ozturan O (2010). Open functional neck dissection: surgical efficacy and electrophysiologic status of the neck and accessory nerve. J Otolaryngol Head Neck Surg.

[17] Cho JG, Lee N, Park MW, Baek SK, Kwon SY, Jung KY, Woo JS (2015). Measurement of the trapezius muscle volume: A new assessment strategy of shoulder dysfunction after neck dissection for the treatment of head and neck cancers. Head Neck.

[18] Ling AO, Toong LY, Ghauth S, Lin NW, Bin Mohamad R (2024). Intraoperative spinal accessory nerve monitoring during neck dissection surgery as a predictor for shoulder dysfunction. Indian J Otolaryngol Head Neck Surg.

[19] Witt RL, Rejto L (2007). Spinal accessory nerve monitoring in selective and modified neck dissection. Laryngoscope.

[20] Lanišnik B, Žitnik L, Levart P, Žargi M, Rodi Z (2016). The impact on post-operative shoulder function of intraoperative nerve monitoring of cranial nerve XI during modified radical neck dissection. Eur Arch Otorhinolaryngol.

